# Complete mitochondrial genome of a predominant parasitoid, *Necremnus tutae* (Hymenoptera: Eulophidae) of the South American tomato leafminer *Tuta absoluta* (Lepidoptera: Gelechiidae)

**DOI:** 10.1080/23802359.2021.1875902

**Published:** 2021-02-12

**Authors:** Xiao-Cao Tian, Xiao-Qing Xian, Gui-Fen Zhang, Cristina Castañé, Jörg Romeis, Fang-Hao Wan, Yi-Bo Zhang

**Affiliations:** a College of Plant Health and Medicine, Qingdao Agricultural University, Qingdao, China; b State Key Laboratory for Biology of Plant Diseases and Insect Pests, Institute of Plant Protection, Chinese Academy of Agricultural Sciences, Beijing, China; c Sustainable Plant Protection Department, Institute for Research and Technology in Agriculture (IRTA), Barcelona, Spain; d Research Division Agroecology and Environment, Agroscope, Zürich, Switzerland

**Keywords:** *Necremnus tutae*, Eulophidae, mitogenome

## Abstract

The complete mitochondrial genome of a predominant parasitoid, *Necremnus tutae* (Hymenoptera: Eulophidae) (GenBank accession number MT916846) is 15,252 bp in length, and contains 13 protein-coding genes (PCGs), 22 transfer RNA genes (tRNAs), 2 ribosomal RNA genes, and an A + T-rich region. The overall base composition is 38.86% for A, 7.14% for C, 8.57% for G, and 45.43% for T, with a high AT bias of 84.29%. ATA, ATT, ATG were initiation codons and TAA and T were termination codons. All the 22 tRNAs displayed a typical cloverleaf secondary structure, except for *trnS_1_* and *trnR* which lacked the dihydrouracil (DHU) arm. Phylogenetic analyses were performed using 13 PCGs showed that *N. tutae* is closely related to *Tenthredo tienmushana*, which in accordance with the traditional classification.

The South American tomato leafminer, *Tuta absoluta* (Meyrick), is native to the western Neotropics. After invading Spain in 2006, it spread rapidly throughout Afro-Eurasia and has become a major threat to world tomato production (Campos et al. 2017; Biondi et al. 2018). Due to the increasing serious insecticide resistance, biological control using Hymenoptera parasitoids was becoming a major management measure of *T. absoluta* (Guedes et al. 2019; Gervassio et al. 2019). *Necremnus tutae* (Hymenoptera: Eulophidae) was a predominant larval parasitoid of *T. absoluta* (Gabarra et al. 2014), however, it was identified as a new species until 2015 (Gebiola et al. 2015). In this study, adult individuals of *N. tutae* were collected from the Institute for Research and Technology in Agriculture (IRTA), Cabrils, Barcelona (N41°30′, E2°22′) in 2019. The specimens were preserved in 95% ethanol and deposited in our laboratory (Department of Biological Invasion, the Institute Plant Protection, Chinese Academy of Agricultural Science) with an accession number CAAS-IPP-DBI-Ntutae2019002.

The complete mitochondrial genome of *N. tutae* (GenBank accession number MT916846) is a circular DNA molecule of 15,252 bp in length, comprising 13 protein-coding genes (PCGs), 22 transfer RNA genes (tRNAs), 2 ribosomal RNA genes (*rrnL* and *rrnS*), and an A + T-rich region (Boore 1999). Twenty-seven genes were encoded on the minority strand (N-strand), while the others were transcribed on the majority strand (J-strand). The overall base composition of *N. tutae* mitogenome is 38.86% for A, 7.14% for C, 8.57% for G, and 45.43% for T, with a high AT bias of 84.29%. The AT-skew and GC-skew of this mitogenome were −0.078 and 0.091, respectively.

Gene overlaps were found at 23 gene junctions and involved a total of 568 bp, and the longest overlap (235 bp) existed between *trnW* and *trnM*. There were five intergenic spacer regions ranging in length from 1 to 10 bp, comprising a total length of 21 bp. The largest intergenic spacer sequence of 10 bp was located between *atp8* and *atp6*. The 22 tRNAs had a total of 1456 bp, and their individual lengths ranged from 61 bp (*trnS_1_*) to 71 bp (*trnT*). All the 22 tRNAs displayed a typical cloverleaf secondary structure, except for *trnR* and *trnS_1_* lost the dihydrouridine (DHU) arm. The lengths of *rrnL* and *rrnS* were 1308 and 771 bp in length, with the A + T contents of 87.23 and 88.2%, respectively. The A + T-rich region was located between *rrnS* and *trnA* with a length of 53 bp.

All the13 PCGs start with typical ATN codons, including two ATAs (*nad1* and *nad5*), six ATGs (*atp6*, *cob*, *cox1*, *cox3*, *nad4*, and *nad6*), and five ATTs (*atp8*, *cox2*, *nad4L*, *nad3*, and *nad2*). Ten PCGs terminate with conventional stop codons (TAA or TAG), and the remaining PCGs including *atp6, nad4*, and *nad6* use a single T as stop codon. According to the relative synonymous codon usage analyses of 13 PCGs, TTA (L), TTT (F), ATT (I), and ATA (M) were the four most frequently used codons. Leucine, isoleucine, phenylalanine, and methionine are the most frequent amino acid of 13 PCGs. Based on the concatenated amino acid sequences of 13 PCGs, the neighbor-joining method was used to construct the phylogenetic relationship of *N. tutae* with 18 other bees with MEGA7 (Kumar et al. 2016). The result showed that *N. tutae* was closely related to *Tenthredo tienmushana* (Figure 1), which agrees with the conventional classification.

**Figure 1.**
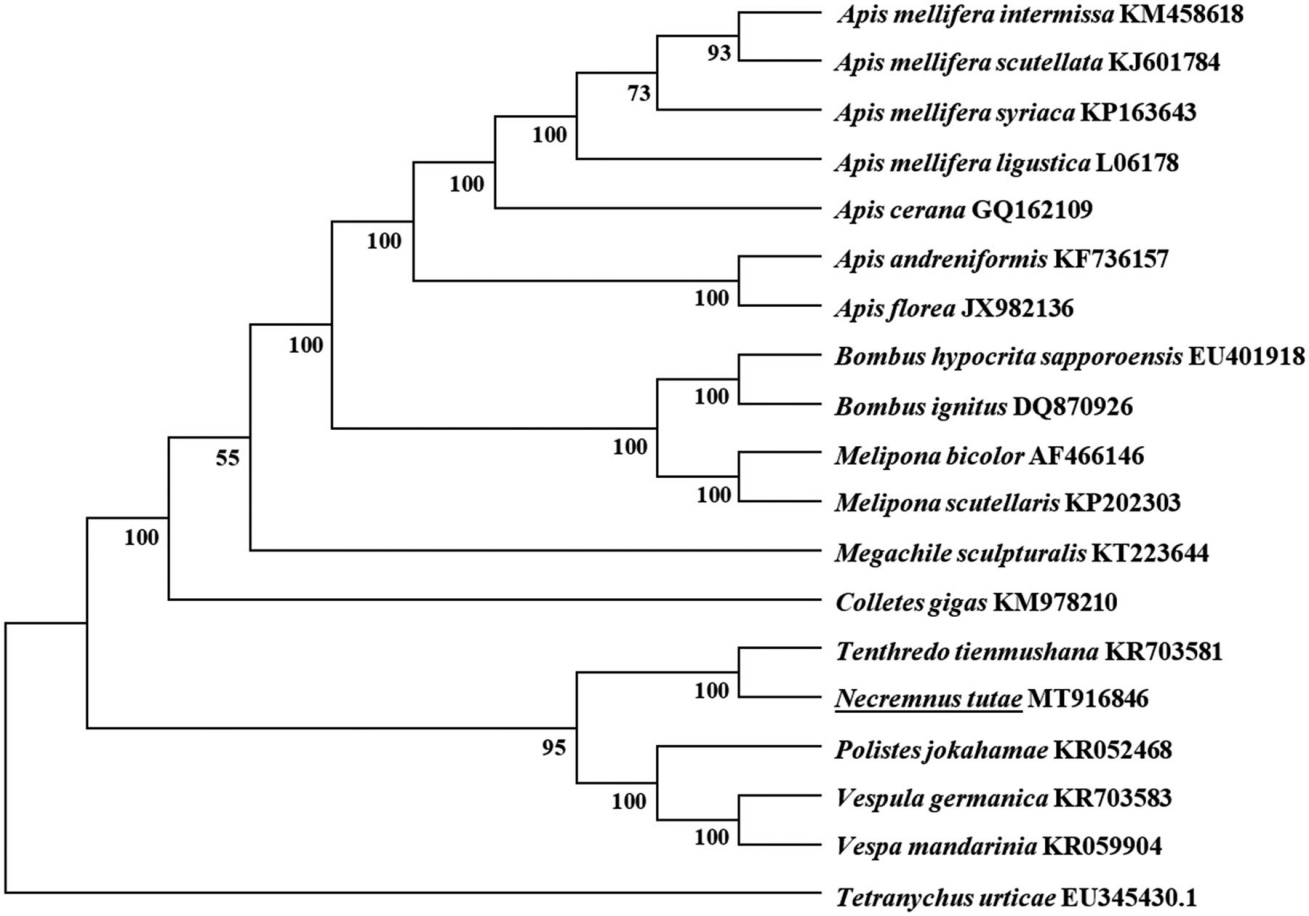
Phylogenetic tree showing the relationship between *Necremnus tutae* and 18 other bees based on neighbor-joining method. *Tetranychus urticae* was used as an outgroup. GenBank accession numbers of each species were listed in the tree. The bee determined in this study was underlined.

## Data Availability

The data that support the findings of this study are openly available in GenBank at https://www.ncbi.nlm.nih.gov/genbank/, reference number MT916846.
